# Electronic Patient Reported Outcome (ePRO) Measures in Patients with Soft Tissue Sarcoma (STS) Receiving Palliative Treatment

**DOI:** 10.3390/cancers15041233

**Published:** 2023-02-15

**Authors:** Silvia Hofer, Leopold Hentschel, Stephan Richter, Veronika Blum, Michael Kramer, Bernd Kasper, Christoph Riese, Markus K. Schuler

**Affiliations:** 1 Department of Neurology, University Hospital Zurich, 8091 Zurich, Switzerland; 2 Division of Psycho-Oncology, NCT/UCC, University Hospital Carl Gustav Carus, Technical University of Dresden, 01307 Dresden, Germany; 3 Clinic and Polyclinic for Internal Medicine I, Sarcoma Center, NCT/UCC, University Hospital Carl Gustav Carus, Technical University of Dresden, 01307 Dresden, Germany; 4 Department of Internal Medicine, Cantonal Hospital Lucerne, 6002 Lucerne, Switzerland; 5 AvenCell Europe GmbH Dresden, 01307 Dresden, Germany; 6 Sarcoma Unit, Mannheim Cancer Center (MCC), Mannheim University Medical Center, University of Heidelberg, 68167 Mannheim, Germany; 7 DTB Gesellschaft für digitale Therapiebegleitung mbH, 07745 Jena, Germany

**Keywords:** soft tissue sarcoma, electronic patient reported outcome, health related quality of life, randomized controlled trial, palliative treatment

## Abstract

**Simple Summary:**

Therapeutic options for advanced soft tissue sarcoma (STS) are limited. Health-related quality of life (HRQoL), along with traditional outcome parameters such as tumor control and toxicity, is one of the most important endpoints for palliative STS treatment. The PazoQoL prospective, randomized, controlled, multicenter study (EudraCT number 2017-003382-10, ClinicalTrials.gov Identifier NCT0373575) was designed to assess the impact of treatment on HRQoL and patient-reported outcomes. Although the study had to be terminated early due to the pandemic, some valuable results were collected on the continuous recording of symptoms over a 9-week period and on patient satisfaction with therapy. Our findings could be translated into clinical practice without much effort and outside of a trial.

**Abstract:**

The PazoQoL prospective, randomized, controlled, multicenter study was designed to continuously assess global health related quality of life (HRQoL) during treatment with pazopanib or physician-preferred chemotherapy over a 9-week period. The questionnaires were completed by the patients at home with great reliability during this time period. Continuous electronic patient reported outcome (ePRO) enabled early detection of the onset of deterioration and timely initiation of countermeasures. The Cancer Therapy Satisfaction Questionnaire (CTSQ) showed high interindividual variability and decline over a 9-week period, whereas the Time Trade-off (TTO) proved to be an efficient method for assessing individual benefit from cancer therapy. In our cohort, the TTO clearly demonstrated that the prolongation of life and the side effect profile of continued therapy were not as satisfactory as expected by patients when starting a new therapy. Although the study had to be stopped early due to the pandemic, our findings could translate into clinical practice without much effort and outside of a trial.

## 1. Introduction

Patient-reported outcome (PRO) measures with the aim to capture health-related quality of life (HRQoL) from a patient`s perspective and without the interpretation of caregivers are key outcome instruments in contemporary clinical trials for cancer treatment [1,2]. The value added from electronic and mobile PRO, hereafter called “ePRO”, includes real-time monitoring, support of therapy-management, lower administrative burden, fewer missing data, and the possibility for immediate interactions [3,4]. Nevertheless, ePRO has not yet been implemented into routine daily practice in soft tissue sarcoma (STS) and oncologists in general demonstrate little familiarity and lack standardization of PROs [5,6]. 

Palliative treatment strategies should aim not only to prolong survival but, more importantly, to control and relieve symptoms, limit disease- and treatment-related morbidity, and preserve the performance of activities of daily living as far as possible. This is particularly important for palliative STS therapies, which offer only modest survival benefits. 

HRQoL in general is a multidimensional construct that takes into account the impact of a person’s health status on their life and can identify unmet needs during treatment and in the follow-up period. It has been previously shown that HRQoL at baseline is a prognostic factor for clinical outcome in various cancers [7,8]. Because HRQoL is based on patient perceptions, relies on self-reflection, and is influenced by impairments, functional status, and social background, all of these measures are useful in discussions with the treating physician as part of shared decision making. 

The PRO questionnaires currently in use, largely lack patients’ views of their expectations and satisfaction with therapy. However, such questionnaires have been developed and validated for different types of cancer, especially for patients receiving intravenous or oral cancer drugs [9,10].

The delicate balance between longer survival and disadvantages of palliative therapy can be captured by the “Time Trade-off” (TTO) method. Two simple and straightforward questions help in the decision-making process for continuing or stopping an ongoing treatment. Patients are asked to rate their preference for quantity versus quality of life, i.e., how much additional survival time a further line of cancer treatment would be worth to them [11,12]. Previous studies that addressed this issue showed that oncologists value prolongation of survival time more than quality of life (QoL) [13]. To date, TTO has rarely been used in sarcoma trials.

Here, we present results of the PazoQoL trial, a randomized, controlled trial (RCT) on QoL in patients with non-adipocyte STS under palliative treatment. The trial was trial designed by the German Interdisciplinary Sarcoma Group (GISG-11; EudraCT number 2017-003382-10, ClinicalTrials.gov Identifier NCT0373575).

## 2. Materials and Methods

The multi-center, longitudinal PazoQoL study allowed patients with several STS subtypes to be included. After progression of one or more lines of systemic STS therapies, patients could be randomized in a 1:1 fashion and allocated to in-label use of the oral agent pazopanib, a selective, multitargeted receptor tyrosine kinase inhibitor of vascular endothelial growth factor receptor 1–3 (VEGFR-1–3), PDGFR-a, PDGFR-b, and KIT or systemic treatment according to investigator’s choice. According to the study plan, 150 patients should have been recruited, 75 in each arm.

**Figure 1:**
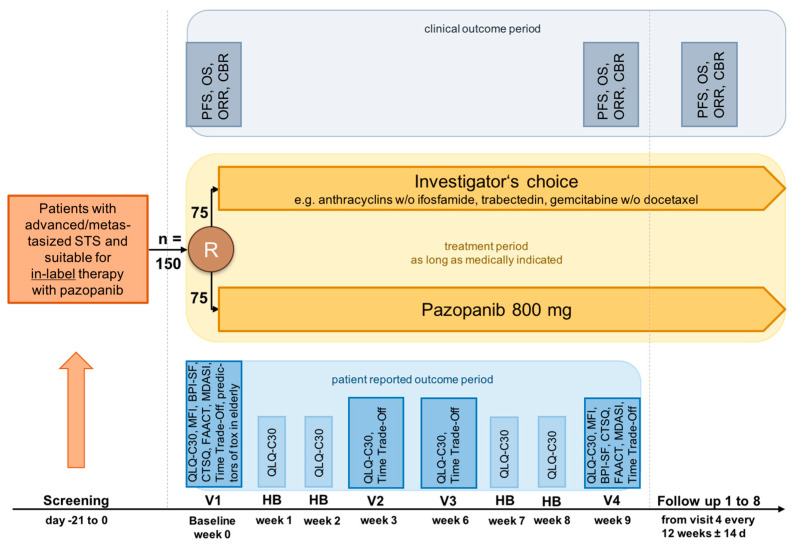
PazoQoL study design. STS, soft tissue sarcoma; V1–4, visit 1–4; HB, home based patient reported outcome measures; PFS, progression free survival; OS, overall survival; ORR, objective remission rate; CBR, clinical benefit rate; QLQ C30, EORTC QoL questionnaire; BPI-SF, brief pain inventory; CTSQ, Cancer therapy satisfaction questionnaire; FAACT, functional assessment of anorexia/cachexia therapy; MDASI, M.D. Anderson symptom inventory; MFI, multidimensional fatigue inventory.

HRQoL as well as other secondary outcome measures, were recorded continuously, i.e., over the first 9 weeks of a new palliative treatment at the times indicated in the protocol (8 in total, Figure 1).

The primary objective of the RCT was the comparison of global HRQoL under treatment with pazopanib or physician-preferred chemotherapy (ChT) after 9 weeks.

Secondary objectives included QoL three times in cycle 1 and cycle 3 (corresponding to weeks 1, 2, 3 and 7, 8, 9, respectively). Other objectives assessed cross-group evaluation of pain, fatigue, and categories such as physical, mental, cognitive, and emotional wellbeing, as well as anorexia/cachexia, both markers of HRQoL. Special attention was paid to parameters of satisfaction with care.

HRQoL was measured applying the EORTC QLQ-C30, a well-validated, extensively used instrument, consisting of 30 items to obtain different domains including five functional scales, symptom scales as well as global QoL. Higher scores (ranging from 0–100) represent higher functioning and global HRQoL, while also describing higher symptom burden [14].

The Cancer Therapy Satisfaction Questionnaire (CTSQ) was used to record satisfaction with therapy (Table 1). The calculation results in a score ranging from 0 to 100 for each domain, with a higher score associated with the best outcome on each domain [9,10].

All participants received tablet-computers with all questionnaires in electronic form to be completed at home. The IT solution Digital Health Management from Compliance Solutions GmbH was used to record and evaluate the patient responses. The tablets with SIM cards (mobile internet) were made available to the respective patients for 9 weeks each to document the diaries.

The questionnaires had to be started by the clinic staff, who trained the patients in their use. After completion of a 9-week patient diary phase, data had to be exported by the clinic staff and then deleted from the device before the tablet had been given to another patient. Access to other functions of the tablets had been blocked for patients.

The PazoQoL study was approved by the Ethics Committee of the State of Berlin (State Office for Health and Social Affairs) and bears the number 17/0390—EK 15 and by the Ethics Committee Northwestern and Central Switzerland (EKNZ) Project ID 2019-00386.

## 3. Results

**Figure 2:**
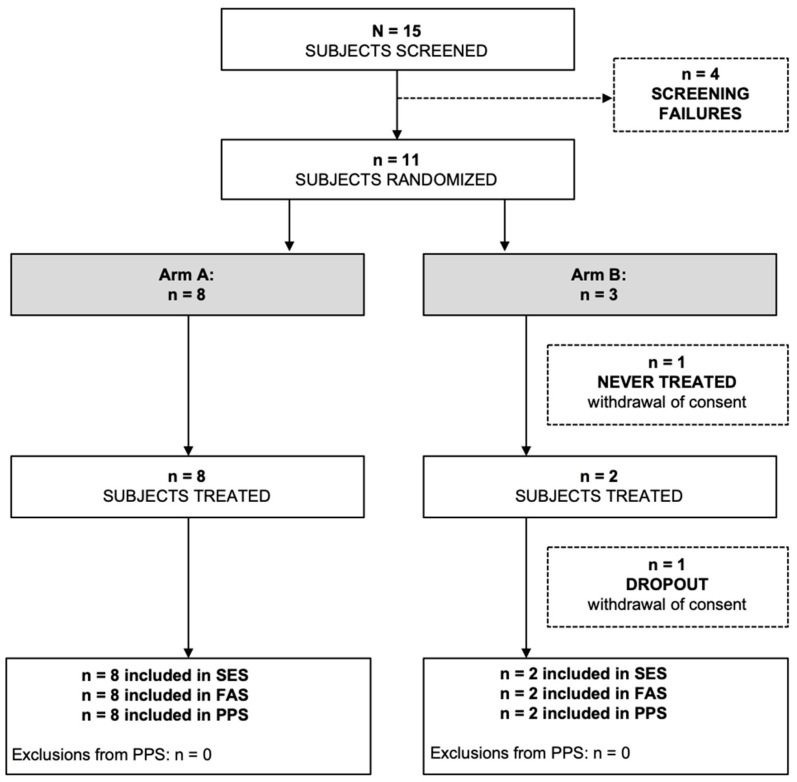
Consort diagram. Arm A, pazopanib; Arm B, physician-preferred chemotherapy; SES, safety evaluation set; FAS, full analysis set; PPS, per protocol set.

The PazoQoL trial was terminated early due to low enrollment during the COVID pandemic and no further funding provided thereafter. Ultimately, only 11 patients could be randomized and 10 of them evaluated (Consort diagram, Figure 2). However, key elements could be exploited despite the small number of patients.

The PazoQoL study was able to demonstrate that a 9-week application of ePRO appears to be sufficient to evaluate therapies for advanced STS in terms of HRQoL and treatment satisfaction.

For the EORTC QLQ30 questionnaire, 92% of the data were complete, and for the TTO and CTSQ questionnaires, the respective rate was 89%. The relatively short assessment period of 9 weeks was therefore associated with a high patient adherence rate.

**Figure 3:**
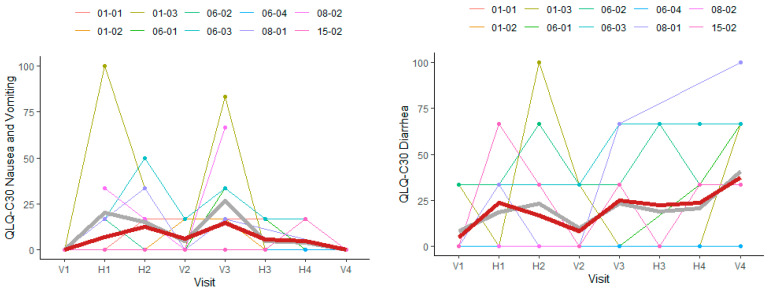
Examples of short-term fluctuations in symptoms.

ePRO enabled tracking of short-term changes of symptoms that varied significantly from patient to patient and over time and therefore did not reflect a general trend (Examples, Figure 3).

(a) Ten individual and mean data for nausea and vomiting, EORTC QLQ-C30 questionnaire. Total mean plotted bold in grey (n = 10), pazopanib arm mean plotted bold in red (n = 8), V1–4, symptoms recorded at regular visits 1–4, H1–4, home based ePRO data (Figure 3 left).
(b) Ten individual and mean data for diarrhea, EORTC QLQ-C30 questionnaire. Total mean plotted bold in grey (n = 10), pazopanib arm mean plotted bold in red (n = 8), V1–4, symptoms recorded at regular visits 1–4, H1–4, home based ePRO data (Figure 3 right).

Close monitoring and continuous ePROs facilitated early detection of incipient deterioration and timely initiation of countermeasures.

The Cancer Therapy Satisfaction Questionnaire (CTSQ) has shown high interindividual variability and a decline over time (Table 2).

The “Time Trade-off” (TTO), an efficient method for evaluating the individual benefit of cancer therapy, yielded the following results: the additional lifetime, a patient would like to gain if he or she is willing to undergo further treatment, increased significantly over time. At the beginning of therapy, it was a median of 24 months; at the end of the study, it was a median of 72 months. When asked how much additional survival time a patient would sacrifice to be symptom-free with continued therapy, the responses were as follows: they would sacrifice a median of 0 months of their lives to be symptom-free (Table 3). However, there were individual patients who would sacrifice some time to be symptom-free. One patient as an example would sacrifice 15 months at visit 1 (baseline) and 12 months at visit 4 (EoT), another patient would sacrifice 1 month at visit 4 after not sacrificing any time at baseline.

## 4. Discussion

PazoQoL has shed light on various aspects for the implementation of ePRO from research to routine and provides recommendations for its use in palliative STS therapy, where individual patient perspectives and preferences are of the utmost interest.

In our study, home-based use of ePRO enabled symptom capture in real time and independent of scheduled visits. A recently published RCT [15] with continuous home measurements was also able to provide a more detailed HRQoL profile and thus, better capture symptom fluctuations.

Satisfaction and expectations with treatment are closely related to decision making and treatment adherence. For this reason, we applied the Cancer Therapy Satisfaction Questionnaire (CTSQ) in our study. To our knowledge, this questionnaire has not been previously used to assess palliative STS therapies. High interindividual variability and decline over time have been observed. Therefore, we consider these personalized statements to be highly relevant and support their implementation in daily clinical practice.

Of particular interest is the TTO, an efficient method of assessing the individual and subjective benefit of a cancer therapy. The results of our cohort clearly indicate that the life extension and side effect profile of continuing therapy were not as satisfactory as expected by patients at the start of a new therapy. Our findings are particularly noteworthy because a detailed and informative discussion with the treating physician took place as a standard procedure in every patient before the start of a next line of therapy. The discrepancy in the different perceptions of the doctor and his patient is a phenomenon that is generally underestimated [13]. Attention to this should be increased and the patient should be given the opportunity to express his concerns in more detail, e.g., to rule out depression as a reason for his current statement.

In order to keep patients’ motivation high to complete the ePRO questionnaires, its application should not exceed a certain time frame, 10–15 min per session might be appropriate (expert opinion). A careful selection of questions covering relevant domains is therefore required. For daily clinical practice, we suggest using a generic and well-established instrument (e.g., the EORTC QLQ-C30 questionnaire) to capture a wide range of symptoms and HRQoL topics and to compare the results with data from ongoing and completed studies. The average time spent completing the QLQ-C30 was reported to be 9 and 7 min before and during treatment, respectively. In our cohort, this time requirement was even lower, at 5.5 and 4 min, respectively. To assess satisfaction with treatment, we suggest including a brief “satisfaction and expectations” questionnaire and the two “Time Trade-off” questions, which take an additional 7–10 min to complete in total (Table 4.).

There will be some costs associated with implementing ePRO in daily clinical practice [16], but these costs are disproportionate to the drug costs that are expected to be recovered as a result. It is noteworthy that regulatory authorities are now paying greater attention to PRO data in their drug approval decisions, in fact, recommendations have already been issued by the U.S. Food and Drug Administration (FDA) and the European Medicines Agency (EMA) [17,18]. One could also imagine that the cost of ePRO could be borne by the pharmaceutical industry in terms of quality of care.

Our RCT is subject to several limitations. Only a small fraction of the planned patients could be recruited due to reasons already mentioned and thus, a majority of the secondary endpoints could neither be reliably evaluated nor could a comparison be made between the two study arms. Nevertheless, our study clearly demonstrates that satisfaction with palliative treatment is a valuable endpoint that can be readily implemented in clinical practice as a suitable tool for shared decision making.

## 5. Conclusions

For daily clinical practice, electronic tools need to be developed further to provide patients with regular reminders and incentives, such as information about their disease, strategies to cope with symptoms, or to enable a prompt way to contact the care team. Data security is a challenge that needs to be harmonized. Since electronic health care systems are being developed worldwide, it is only a matter of time before the infrastructure for ePRO will be widely available and only the contents of choice need to be filled in.

HRQoL, along with traditional outcome parameters such as tumor control and toxicity, is one of the most important endpoints for palliative STS therapies.

## Figures and Tables

**Table 1:** Cancer therapy satisfaction questionnaire (CTSQ) domains. https://docplayer.net/51309772-Administration-and-scoring-guide-for-the-cancer-therapy-satisfaction-questionnaire-ctsq.html (accessed on 12 February 2023).

CTSQ Domains	Content of Items
Expectations of therapy	Return to normal life. Get rid of cancer. Prevent cancer from coming back. Stop cancer from spreading. Help you to live longer
Feeling about side effects	Cancer therapy (CT) limited daily activities. Upset about side effects. Taking CT as difficult as expected. Were side effects as expected
Satisfaction with therapy	Worth taking even with side effects. Think about stopping CT. How worthwhile was CT. Benefits meet expectations. Satisfaction with form of CT, Satisfaction with recent CT. Would you take the CT again

**Table 2:** Results Cancer Therapy Satisfaction Questionnaire (CTSQ). Given numbers are mean/SD and (min–max); EoT, end of treatment; Score ranging from 0–100 for each domain, with a higher score associated with the best outcome.

Domains		All Patients, n = 10	Pazopanib Arm, n = 8
Expectations of therapy	Baseline EoT	43.89/28.7 (0–95) 36.25/31.48 (0–100)	37.86/29.56 (0–95) 35.71/33.96 (0–100)
Feelings about side effects	Baseline EoT	43.06/22.41 (12.5–75) 37.50/29.32 (0–87.5)	41.96/25.19 (12.5–75) 30.36/22.94 (0–62.5)
Satisfaction with therapy	Baseline EoT	17.00/4.21 (10–23) 15.00/6.21 (8–25)	16.43/4.35 (10–23) 13.57/5.09 (8–22)

**Table 3:** Results Time Trade-off (TTO). Given are median (SD), EoT, end of treatment ChT, chemotherapy.

Parameter		All Patients, n = 10
Extra time for ChT (months)	Baseline EoT	24 (12–72) 72 (54.5–72)
Time for being symptom-free (months)	Baseline EoT	0 (0–0) 0 (0–0.25)

**Table 4:** Proposal for ePRO in daily clinical practice for advanced STS. CTSQ, Cancer Therapy Satisfaction Questionnaire, QoL, quality of life, STS, soft tissue sarcoma.

	Captures	Questions	Time Spent
EORTC QLQ-C30	Physical, psychological & social functions	30	4–5 min
CTSQ	Satisfaction with therapy	16	5–10 min
Time Trade-off	QoL	2	2 min

## Data Availability

The data underlying this article will be shared on reasonable request to the last author M.K.S.
